# A holistic exploration of the psychosocial, environmental, neurobiological, and individual factors influencing children’s food choices: a narrative review

**DOI:** 10.3389/fnut.2025.1645293

**Published:** 2025-09-25

**Authors:** Laila Albardan, Carine Platat

**Affiliations:** Department of Nutrition and Health, College of Medicine and Health Sciences, United Arab Emirates University, Al Ain, United Arab Emirates

**Keywords:** psychosocial factors, environmental influences, brain mechanisms, individual differences, eating behavior, child

## Abstract

Children’s food choices often last into adulthood, playing a crucial role in their long-term health outcomes. Adopting unhealthy eating habits can lead to obesity and chronic diseases. To understand the complexities of children’s eating behaviors, there is a need for a comprehensive examination of many factors that affect their food choices. This narrative review examines the interplay of psychosocial, environmental, neurobiological, and individual-level factors that influence children’s dietary choices. Through a thorough literature search, this review highlights how early dietary habits are predominantly influenced by parental modelling and the home environment. These factors work alongside genetic traits and changes caused by maternal nutrition during pregnancy and breastfeeding. Broader factors, such as peer influence, food marketing, school policies, and food availability, can either support or hinder the development of healthy eating habits. Moreover, biological factors, including gut bacteria, hormones that regulate appetite, and the brain’s reward system, significantly influence children’s food preferences. Unlike earlier reviews that examined these influences separately, this review presents a broader perspective on the interplay between these areas. The findings emphasize the need for a multi-level approach that combines individual, family, and community strategies to inform future research and policy aimed at promoting sustainable and healthy eating behaviors among children.

## 1 Introduction

Healthy eating is crucial for children’s growth, development, and overall wellbeing. Adequate nutrition promotes physical growth, supports cognitive development, and enhances immune function, laying the foundation for lifelong health (1). On the other hand, poor dietary habits during childhood can lead to growth delays, developmental challenges, and long-term health issues (2). Unhealthy eating patterns are also associated with the rising rates of chronic diseases in later life (3). Research shows that eating behaviors formed in childhood often tend to continue into adulthood, increasing the risk of chronic conditions such as obesity, type 2 diabetes, cardiovascular diseases, and mental health issues (4, 5). The World Health Organization reports that, in 2022, over 390 million children and adolescents aged 5–19 years were considered overweight, with 160 million being obese. The rates of overweight and obesity rose significantly from 8% in 1990 to 20% in 2022 (6). Childhood obesity impairs cognitive function (7), increases the likelihood of developing adulthood obesity (8), and raises the risk of cardiovascular diseases in adulthood (9). Therefore, understanding the factors that influence children’s dietary choices is crucial for designing effective interventions that promote sustainable, healthy eating habits from an early age.

Studies on children’s eating habits reveal both external and internal factors that influence their food choices (10). Early research indicates that children possess a natural regulatory mechanism for managing food intake, demonstrated by their ability to compensate for calorie deprivation and feel full after consuming certain foods (4). On the contrary, some studies suggest that children may sometimes eat even when they are not hungry (11), implying the influence of other psychosocial and environmental factors. Bronfenbrenner’s ecological systems theory shows how multiple layers of influence, from personal to societal, impact behavior. This model includes psychosocial and environmental factors such as parental and peer influences, socioeconomic status, food availability, and media exposure (12). However, the Socio-Ecological Model, which is based on Bronfenbrenner’s ecological systems theory, is the most used framework in public health. It highlights how individual, interpersonal, organizational, community, and policy-level factors work together to shape dietary choices (13, 14). Still, some factors like neurobiological (2, 15) and genetic and epigenetic influences (16–19) are not fully incorporated into this framework, despite their important role in influencing dietary choices.

A complex interplay between psychosocial, environmental, neurobiological, and individual factors affects children’s dietary choices. Psychosocial factors include parental and peer influences, as well as cultural norms (4). Environmental factors cover food availability, marketing, and school settings (20). Neurobiological factors, such as brain development, dopamine system activity (15), hormone regulation (2), and the gut-brain axis (21), significantly influence children’s sensitivity to food rewards. Individual factors are influenced by innate food preferences (22), emotional states (103), as well as genetics and epigenetics (19). Together, they shape long-term dietary preferences (23). While these areas have been widely researched, they are frequently studied in isolation, which restricts our comprehensive understanding of the whole picture.

This review aims to describe psychosocial, environmental, neurobiological, and individual determinants of children’s food choices, to highlight how they interact, and discuss implications for nutrition recommendations, interventions, and policy development.

This review is grounded in existing frameworks and proposes a hybrid model that integrates psychosocial, environmental, neurobiological, and individual determinants to guide the analysis. Each determinant and its interactions are examined to support the development of nutrition recommendations, interventions, and policy, identify research gaps, and propose future directions.

## 2 Existing theoretical frameworks and rationale for the hybrid model

**Figure 1:**
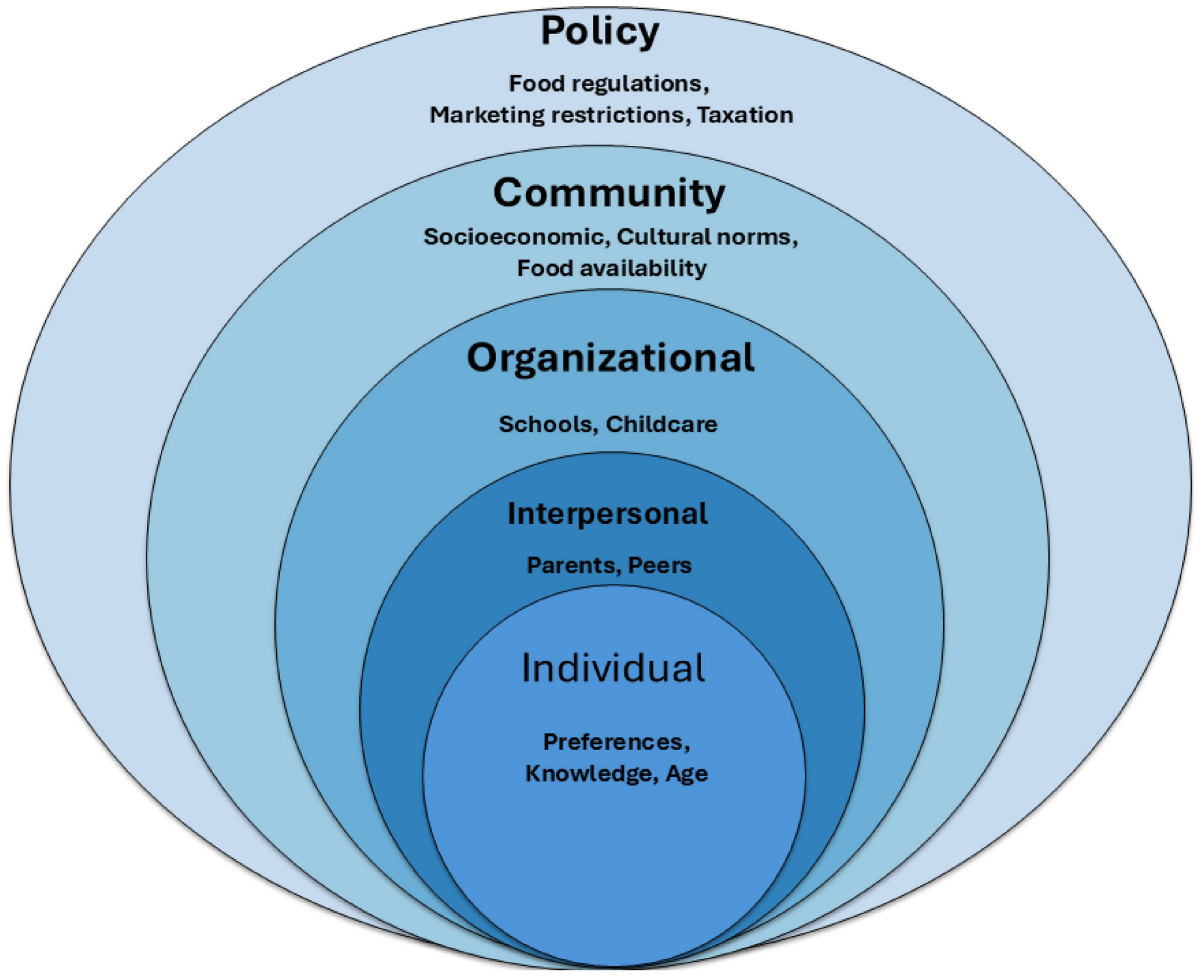
Socio-ecological model (SEM) adapted for children’s food choices.

Several theoretical frameworks have been used to examine children’s eating behaviors. The Socio-Ecological Model (SEM) is the most widely applied (Figure 1). It emphasizes how individual, interpersonal, organizational, community, and policy-level factors shape dietary practices (14). The Biopsychosocial Model adds biological predispositions and physiological processes, alongside psychological and social influences (24). Cognitive-behavioral perspectives, such as Social Cognitive Theory (SCT) (25) and the Theory of Planned Behavior (TPB) (26), focus on observational learning, perceived norms, and intention-driven behaviors.

While each framework offers useful insights, they also have key limitations. The SEM, though thorough, often downplays the role of neurobiological and genetic factors in food choice. The Biopsychosocial Model tends to view influences as separate rather than interconnected. SCT and TPB focus on psychosocial and cognitive aspects but overlook wider systemic and biological factors. As a result, these models give only a limited view of the complex interactions that shape children’s food preferences and behaviors.

To address these gaps, this review presents a hybrid framework that places children at the intersection of psychosocial, environmental, neurobiological, and individual factors. This combined view acknowledges that children’s dietary behaviors are influenced by their innate characteristics, including taste sensitivity, genetics, and emotional state. They are also influenced by family and peer relationships, environmental factors such as availability, marketing, and policy, as well as neurobiological processes, including reward pathways, hormonal regulation, and the gut-brain axis. By combining elements from existing frameworks, the proposed hybrid model offers a more complete way to interpret evidence and guide the structure of this review.

The SEM reveals different levels of influence on children’s eating behavior, ranging from personal factors to broader policy issues. This framework acts as the reference model for the current review.

## 3 Methodology

To collect relevant literature for this narrative review, an extensive search of scientific databases, including PubMed, Scopus, and Google Scholar, has been conducted. Literature published from the early 2000s through 2025 was considered, with an emphasis on recent (post-2010) studies to capture up-to-date findings. We used combinations of keywords such as children, food choice, food preferences, dietary behavior, parental influence, peer influence, *school* food environment, socioeconomic status, media, marketing, culture, brain reward, gut microbiome, hormones, emotional eating, genetics, and epigenetics. Inclusion criteria encompassed English-language peer-reviewed publications (original research studies, systematic and narrative reviews, and meta-analyses) that examined one or more factors influencing food choices in pediatric populations (aged 18 years or younger). We selected articles that addressed psychosocial (family, peers, culture, etc.), environmental (food availability, school, media, policy), neurobiological (brain development, hormones, microbiota), or individual (preferences, genetics and epigenetics, and emotional factors) determinants of eating behavior. Key references cited within reviewed articles were also reviewed to identify additional relevant sources.

## 4 Individual factors influencing children’s food choices

Various individual factors, including genetic and epigenetic influences, innate and learned food preferences, and emotional factors, affect children’s food choices. These factors are intrinsic to the child and interact with external psychosocial and environmental influences to shape dietary behaviors. This section examines the impact of genetic and epigenetic factors, food preferences, and emotions on children’s food choices.

### 4.1 Food preferences

Children’s food preferences significantly affect their nutritional choices. They are born with a natural liking for sweet and salty tastes and an aversion to bitter and sour flavors (22). These preferences likely evolved to help them identify energy-rich foods (108) and avoid potentially harmful ingredients (22). The preference for sensory-rich foods activates the brain’s reward and sensory pathways, reinforcing the desire for immediate pleasure and possibly leading to overeating and weight gain (109). Nonetheless, cultural practices, family environments, and repeated exposure influence and modify these innate tastes (27). For example, children can learn to enjoy foods they initially disliked, such as certain vegetables, after multiple tastings. Early exposure to a variety of flavors, such as through maternal diet during pregnancy or repeated offerings during childhood, can expand a child’s palate (18, 28). Conversely, frequent exposure to sugary snacks may heighten a child’s preference for sweet foods (29). Therefore, while biology provides children with an initial set of likes and dislikes, experiences play a crucial role in shaping their food preferences over time.

### 4.2 Genetic and epigenetic factors

Behavioral genetics research has demonstrated that nearly all aspects of child behavior, including eating behaviors, have a genetic basis (19). Food preferences in a large pediatric twin sample cohort discovered that genetic factors had a more substantial influence on preferences for nutrient-dense foods like vegetables (54%), fruit (53%), and protein (48%), while shared environmental effects were more prevalent for energy-dense foods like snacks (60%), starches (57%), and dairy (54%) (30). It is well established that children’s biologically determined predilection for sweet flavors drives them to breast milk and has analgesic effects. At the same time, their increased aversion to bitter tastes, which diminishes by mid-adolescence, shields them from toxins (31).

Modern genomic studies have begun identifying specific gene variants with single-nucleotide polymorphisms (SNPs) associated with taste and eating behaviors. For example, a variant in *TAS1R2* (rs7513755), which affects sweet taste receptors, and a variant in *OR10G3* (rs34162196), which affects olfactory receptors, have been linked to higher intake of sugary foods and a stronger sweet tooth in children. These genetic variants may also influence children’s eating choices by predisposing them to favor sweeter foods, as parental genetics can influence offspring’s attributes. Notably, genetic heritage does not act in isolation; it frequently interacts with environmental exposure. For example, a mother carrying “sweet preferring” genetic variants might also have a diet higher in sugary foods, exposing the child to those flavors prenatally or via breast milk. Indeed, studies have found that maternal dietary habits during pregnancy can modulate the child’s later food preferences (32). This finding is consistent with other research indicating that early exposure, maternal diet during pregnancy, and genetic predisposition influence children’s food preferences and eating habits (19).

However, although genetic determinants are significant, epigenetic mechanisms—modifications in gene expression without altering the underlying DNA sequence resulting from environmental influences—also play a crucial role (19, 23), which are influenced by maternal nutrition during pregnancy and lactation (16–18). For instance, caloric restriction and diets rich in fat or typical restaurant meals during these stages can cause an increase in appetite (hyperphagia) in offspring. Similarly, exposure to starvation or limited intrauterine growth has been associated with a later preference for high-fat or sugary foods (16). One study found that high adherence to a Mediterranean diet during pregnancy was associated with improved behavioral outcomes in offspring (33). Additionally, research indicates that high maternal vegetable intake during pregnancy and lactation is associated with stronger vegetable preferences and increased consumption in their children (18, 28). Combining high vegetable intake with extended breastfeeding appears to further increase vegetable consumption in offspring (28). Overall, this evidence highlights the significant role of pregnancy and lactation in shaping lifelong dietary preferences, with maternal diet influencing children’s eating habits for up to a decade post-birth.

### 4.3 Emotions

Emotions significantly influence children’s food choices, often driving behaviors such as emotional eating or food avoidance. Emotional states, including happiness, sadness, stress, or boredom, are known to significantly impact food choices (103). Children often use food to regulate their emotions, with comfort foods or high-calorie, palatable foods particularly appealing during negative emotional states (104). Evidence suggests that negative emotional states are linked to unhealthy foods, while positive emotional states are associated with healthier food choices. This pattern of eating to cope with emotions (especially negative ones) is a known risk factor for the development of obesity and binge eating disorder (105). However, overweight and obese children also have a stronger emotional response to food, leading to a preference for less healthy foods (10, 34). Therefore, this creates a challenging cycle: using junk food to self-soothe can lead to excessive calorie intake and weight gain, which in turn may cause further emotional distress and preserve the reliance on food for comfort.

Individual differences are also important, as unpleasant emotions might reduce appetite in some children, or they can cause children who already associate emotions with food to eat more disinhibited or hedonistically (35). Although emotional eating in children has been linked to increased intake of energy-dense foods, most evidence comes from self-report questionnaires, which are subject to potential bias. There is a lack of longitudinal data and conflicting evidence regarding the direction of this link, as it remains unclear whether a rise in emotional eating behaviors is preceded by poor food quality.

## 5 Psychosocial factors influencing children’s food choices

Numerous psychosocial factors strongly influence the dietary choices of children. From a developmental perspective, a child’s eating behaviors are shaped by exposure and socialization from a very young age (101). The family environment is typically the child’s first context for learning about food—parents and caregivers introduce not only the available foods but also attitudes toward those foods and mealtime practices that reflect cultural or familial norms. As children grow older, influences broaden beyond the home: peers, teachers, media figures, and cultural context all contribute to shaping food preferences and habits (4, 102). In this section, we discuss key psychosocial determinants, including parental influence, peer influence, *and* cultural influences on children’s food choices.

### 5.1 Parental influence

Several studies have shown that parents influence their children’s eating habits in various ways. Providing children with access to healthy meals at home can help them develop a preference for such foods (27, 36).

For instance, the HOME Plus study showed that parental role modeling of fruit and vegetable consumption had a positive correlation with the nutritional intake and preferences of children aged 8 to 12 years old. Children who observed their parents’ consuming vegetables at snack time and salad at dinner were significantly more likely to meet daily fruit and vegetable recommendations. Children who met the recommended intake were also more likely to have parents who reported modeling fruit consumption during snack time (37). However, it is still unclear whether parents or children are better at reporting behaviors, as both can provide invaluable information. These findings demonstrate that children are aware of their parents’ eating habits and that parents can help them develop healthy eating habits by modeling good eating behaviors, particularly during dinner and snack times. Similarly, another study observed data from 1,435 families in eight European countries and found that children’s consumption of nutritious meals was significantly influenced by their home food environment, especially for children under 11 years of age (38). Although the home environment has been observed to be the primary driver of healthy meal intake, genetic variants have not been considered. Parental modelling concept aligns with Social Cognitive Theory, which suggests that behaviors are learned through observation, imitation, and modeling within a social context (39). Parents act as role models by providing healthy foods at home, indirectly teaching children what constitutes a healthy diet.

However, parental strategies for guiding food choices are complex and multifaceted (40). Rewarding children for eating certain foods may encourage healthier choices but can also have adverse long-term effects on eating behavior if overused (41). For instance, Jansen et al. (42) have observed that parents who use food as a reward are associated with their children eating more emotionally and becoming more finicky about their food over time. This suggests that giving children sweets or snacks as rewards may lead them to connect food with emotions and make them less likely to consume healthy meals. Nonetheless, no consistent correlations were identified between the utilization of food as a reward and children’s satiety responsiveness or BMI/weight status. This may be attributed to the opposing effects of selective eating (associated with lower weight) and emotional overeating (associated with higher weight), as well as the complex impact of food rewards on adiposity (42). However, the “food as reward” concept relied on merely two Child Food Questionnaire items, hence constraining external validity.

Different parenting styles, such as authoritative or permissive, influence children’s eating habits. Studies found that authoritative parenting, marked by a balance and without being patently restrictive, is correlated with children’s lower BMI and healthier eating habits (43, 44), while strict control may result in weight gain and an obsession with items that are forbidden (4, 44, 45). The 1,492-child Quebec Longitudinal Study of Child Development found that lower soft drink intake and higher fitness levels were associated with a positive family environment characterized by less pressure to eat. Children who felt less pressure from their parents drank significantly fewer soft drinks and were more fit (46). Thus, this implies that children are more likely to form healthy eating habits when parents establish a welcoming and less restrictive food environment at home (40, 45). Thus, parents act as role models for their children, influencing their motivations, eating habits, and even their perception of their bodies (27, 45, 47). According to Bronfenbrenner’s Ecological Systems Theory, which emphasizes the interaction between a person’s development and the systems in their environment, parental control over nutrition should be balanced to promote autonomy while providing guidance (12).

Although many studies have found that parental modeling and authoritative eating have a positive impact on children’s consumption of fruits and vegetables, the majority of this data comes from countries with higher incomes, making it difficult to generalize the findings to low- and middle-income settings where cultural norms and eating environments differ significantly. Several observational studies report similar associations [e.g., (38, 46)], but these designs cannot establish causality and may be confounded by parental health consciousness or socioeconomic status.

### 5.2 Peer influence

Peers can also significantly influence the type and amount of food children choose, as they often imitate the eating habits of their peers and may modify their choices to conform to social expectations. Peers can lead children toward less healthy options, such as high-energy, low-nutrient foods, but can also positively encourage healthier habits (48). Therefore, this demonstrates the importance of promoting positive peer modeling, particularly in obesogenic environments where unhealthy, energy-dense foods are normalized.

An illustrative finding by Greenhalgh et al. (106) showed that the power of peer modeling can depend on the child’s age. In that study, for children aged 5–7, seeing a peer positively model eating vegetables (e.g., expressing liking for them) helped counteract the impact of another peer rejecting vegetables – in other words, positive peer influence mitigated negative modeling in this age group. However, in younger children (ages 2–4), the same effect was not observed; their immediate preferences or parental cues may have a greater influence on toddlers and preschoolers than peer behavior. This suggests that as children grow, peers increasingly become significant models for behavior, which is consistent with developmental theories. The influence of peers on food choices aligns with the Theory of Planned Behavior, specifically the component of subjective norm (26): if a child perceives that “everyone else” is eating a certain way, they feel social pressure to do the same. Therefore, fostering environments where healthy eating is the norm among peers (for instance, school policies that encourage all students to have fruit as snacks, or healthy cooking clubs) can be a powerful tool.

It is worth noting that research on sibling influence is limited; however, older siblings are likely to act as a unique group of peers. Some evidence suggests that older siblings can influence younger siblings’ food choices (both positively and negatively) (49), yet this area is still not well-studied. In general, the ways peer influence works—such as peer pressure, the desire for social acceptance, and group norms—need more research in the context of eating. Understanding these factors could help develop interventions that influence peer networks to foster healthier dietary habits.

### 5.3 Cultural influences

Cultural factors play a significant role in shaping dietary preferences (50, 51). Children’s food choices and eating habits are influenced by various aspects, including the social importance of meals, cultural views on food, and traditional nutritional practices (50). For example, in many cultures, meals are seen as occasions to share, which strengthens family and social bonds. These cultural views on food can affect children’s eating behavior from a young age, either supporting or hindering the development of healthier eating patterns (51).

In multicultural settings, children might experience different dietary practices. This exposure can either expand or limit their food preferences based on their surroundings (51). As global migration rises, the mixing of various cultural food norms becomes clearer. This raises concerns about how these diverse influences impact children’s eating habits over the long term (52).

## 6 Environmental factors influencing children’s food choices

In addition to family and peer dynamics, environmental factors significantly influence children’s food choices. The environment includes the settings where children live and make food decisions. This encompasses schools, media exposure, socioeconomic conditions, and policy environments. These factors affect which foods are available or affordable and the cues children get about food. This section examines the impact of the school food environment, media and marketing, socioeconomic status, food affordability, and broader environmental and policy factors.

### 6.1 School food environment

Research has shown that access to resources and eligibility for free school meals affect children’s food choices in elementary schools (53). de França et al. (54) also found that fast food and convenience stores dominate the school food environment, providing unhealthy options. While this setting may contribute to obesity, most food is consumed at home (54). Therefore, further research is needed to investigate the impact of the home and school environment on children’s eating habits and to determine which environment has a greater influence.

Nevertheless, many school-based interventions have been implemented and have been found to influence children’s food choices. A systematic review evaluated interventions on school food policy and their effects on children’s diets, finding that competitive food standards decreased the consumption of sugary drinks and unhealthy snacks. In contrast, policies promoting healthy foods increase the consumption of fruits and vegetables. Although school meal standards reduced the amount of fat and sodium consumed, they had no discernible impact on overall calorie intake or body weight (20). These findings are consistent with other systematic reviews of school-based nutrition interventions, which have shown promise in improving children’s dietary intake, particularly by reducing dietary fat and increasing fruit and vegetable consumption. Although the results were less consistent, some studies also suggest advantages in lowering calorie intake, reducing consumption of sugar-sweetened beverages, and avoiding unhealthy snacks (55).

However, differences in how schools produce, supply, sell, or serve food, as well as policy-based nutritional recommendations, could all contribute to unmeasured heterogeneity. This is because educational institutions and schools differ both within and between countries. Additionally, a lack of reported information about socioeconomic factors is observed in most studies, which could impact the effectiveness of some interventions. Finally, most studies originate from wealthy Western countries, which necessitate research in lower-income countries.

While experimental research on modifying the food environment and providing nutrition education in school settings shows promise, the long-term sustainability and cost-effectiveness of these approaches remain underexplored, with a particular emphasis on poorer countries.

### 6.2 Media and marketing influence

Beyond the school environment, media and advertising also play a pivotal role in shaping children’s food preferences. Advertising and marketing strategies targeting children have a significant influence on their perceptions and preferences, often promoting high-calorie, low-nutrient foods (56–59). Entertainment media can significantly influence children’s dietary preferences through persuasive cues, including affective and cognitive cues (34, 57). Exposure to typical marketing cues can lead children to prefer branded, nutrient-poor foods and to pressure parents into purchasing them. In contrast, the inclusion of healthy foods in cartoons, particularly when combined with affective signals, can enhance children’s appraisals and fruit selections.

However, the children’s susceptibility to media influences and the impact of cognitive cues can vary with age, BMI, and media literacy (34). Substantial evidence exists connecting marketing exposure to children’s food preferences, but observational studies often restrict causal conclusions. While experimental research reveals immediate impacts on product choices, the long-term significance of these effects, particularly across different cultures and age groups with varying media rules, remains uncertain.

### 6.3 Socioeconomic status and affordability

Socioeconomic status significantly affects food choices, which impacts the availability of healthy meals (60, 61). People with lower socioeconomic status often have poor diet quality. They tend to eat more foods that are high in energy but low in nutrients (62). This issue worsens due to limited access to good food options, financial difficulties, and a lack of nutrition education. Similarly, Gautam et al. (63) demonstrated that children from low socioeconomic backgrounds are more likely to engage in unhealthy behaviors, such as consuming high-energy-dense foods. In contrast, children from higher socioeconomic status usually have healthier habits. They are more likely to eat breakfast regularly, consume more fruits and vegetables, and maintain a nutritious diet.

Lower socioeconomic status tends to be associated with limited access to nutritional education. This leads to a lack of awareness about the importance of a balanced diet. Additionally, children of younger mothers with less education and limited understanding of nutrition had the highest intake of unhealthy foods (64, 65). These results suggest a need for targeted nutrition programs that consider sociodemographic factors and the level of nutritional knowledge.

### 6.4 Broader environmental and systemic factors

In addition to individual, media, and school-level influences, children’s food choices are significantly influenced by broader environmental factors, including national policies, urbanization, and the availability of grocery stores. Food policies regulating taxation on unhealthy foods can change consumption patterns on a larger scale. For example, countries that have implemented sugar taxes have reported a decline in the purchase of sugary beverages (66). Urbanization also affects food accessibility. It has been observed that urbanization is positively associated with increased consumption of meat, fruits, dairy products, and home-prepared meals while being negatively linked to the consumption of vegetables and sweet foods. These effects are more significant among households with lower socioeconomic status (67).

Although the availability of healthy foods is a consistent predictor of healthier dietary intake, most studies are cross-sectional and conducted in urban, high-resource settings. Findings may not generalize to rural or low-resource contexts where food environments are more constrained.

## 7 Neurobiological factors influencing children’s food choices

Neurobiological factors, mainly those related to brain development and reward systems, significantly influence children’s food choices. The brain’s structural changes caused by obesity can also influence decision-making and increase impulsivity toward food (15, 68). Additionally, food preferences are shaped by the gut-brain axis, which controls reward and hunger. Disruptions in this axis can result in unhealthy eating habits (21). Children’s food choices are also affected by hormones that manage appetite, cravings, and reward-based eating. Imbalances in these hormones can lead to overeating and obesity (2, 69–72).

### 7.1 Brain development and reward systems

Regions that are responsible for reward and decision-making change significantly during childhood, which affects how children respond to food (15). The dopamine system is crucial for processing rewards (73), and it is very active in childhood (15). This system encodes the positive reinforcement associated with enjoyable experiences, such as eating tasty foods.

Children’s brains react more intensely to food signals in areas linked to motivation and reward compared to adults. This difference is due to ongoing brain development and less developed self-control. When children see unhealthy foods, their brains show more vigorous activity in areas related to reward processing and self-control. This can lead to unhealthy eating habits and weight gain (15).

**Figure 2:**
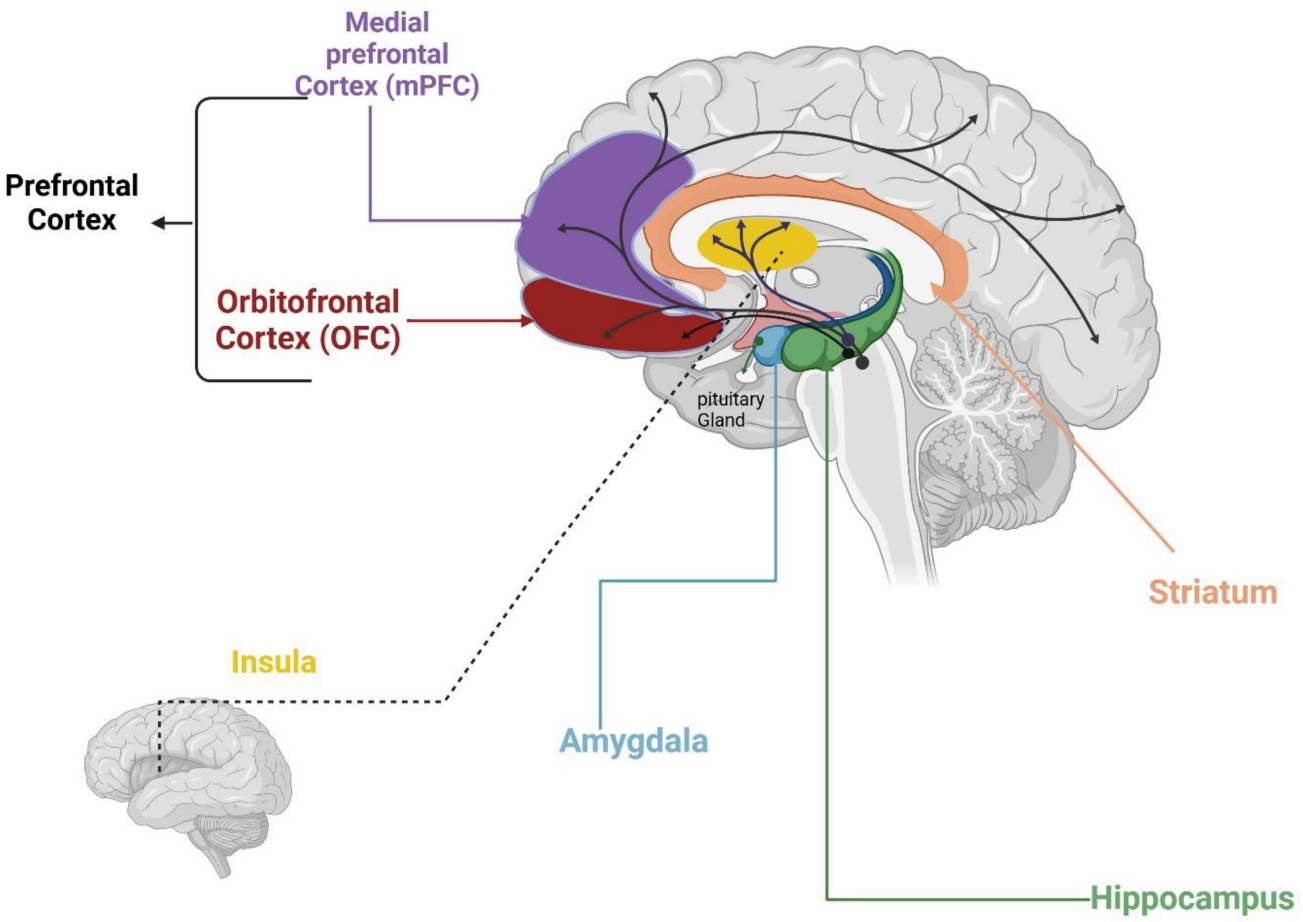
Brain regions activated by food cues. Created in BioRender. Hilary (107) (https://BioRender.com/p91v928).

In children, several brain regions are involved in the reward system in response to food cues (Figure 2), including the amygdala, striatum, orbitofrontal cortex (OFC), Medial Prefrontal Cortex (mPFC), and insula (10, 74). These regions collaborate to form a network that evaluates the emotional and rewarding aspects of food. The amygdala processes emotional responses to food stimuli. It affects children’s motivation to eat by determining the appeal of food (75). The OFC combines sensory input—including appearance, taste, and smell- with reward value (74, 76), increasing activity when exposed to high-calorie and appealing meals (10). Besides processing internal signals like hunger and fullness, the insula also responds to external sensations of food, such as smell and taste. Therefore, the insula is key in merging sensory, emotional, and cognitive information, which affects how children choose what to eat (77). The striatum enhances responses to tasty foods, thereby boosting motivation for eating (10). The mPFC controls decision-making and inhibitory control, but its activation decreases when children face unhealthy, high-calorie options. This reduction leads to poor self-control and a higher risk of overeating, especially in children at risk for obesity (10). These regions work together to create a reward system that balances sensitivity to rewards and self-control, ultimately shaping children’s food choices and preferences.

The reward sensitivity in these brain regions is strong in children. This leads to a clear preference for foods that offer immediate pleasure, especially those high in sugar and fat. This increased reward sensitivity has two main effects. First, it helps children learn to enjoy eating. Nevertheless, it also makes them more likely to overeat of tasty foods, which can lead to unhealthy eating habits (15). The hippocampus influences food choices by recalling associations with pleasure and satisfaction. It holds memories, particularly of past eating experiences (10). Secondly, since the prefrontal cortex, responsible for self-regulation, is still developing in children, it limits the child’s ability to regulate responses to food cues, making them more susceptible to immediate sensory rewards over long-term health considerations (15, 78).

Furthermore, studies demonstrate a reverse association between childhood obesity and brain structure (97). The brain regions responsible for pleasure, reward, decision-making, and self-regulation can all be affected by obesity-related structural changes (68). Research indicates that obese children exhibit reduced white matter integrity and decreased grey matter in many brain regions. Additionally, they show different activation levels in specific brain regions associated with processing food signals, such as the amygdala and insula, which heighten sensitivity to food cues and impair impulse control (15). These differences make it more challenging to regulate food intake, thereby increasing the risk of overeating and subsequent weight gain (15, 68).

Neuroimaging research indicates that children respond more strongly to appealing food cues in areas associated with reward and motivation. For example, unhealthy foods elicit stronger activation in areas involved in reward, motivation, and memory in children compared with adults, with children at higher BMI exhibiting reduced medial prefrontal and orbitofrontal reactivity to food cues, suggesting weaker inhibitory control (79, 80), with boys showing stronger hippocampal and visual responses than girls (79). Functional MRI studies further show that children at high risk for obesity (e.g., those carrying obesity-predisposing gene variants) exhibit greater activation in reward-related brain regions, such as the insula, when viewing high-calorie food cues. Thus, this heightened neural responsivity may predispose at-risk children to stronger cravings and impulsive overeating (81).

The environmental context also shapes neural responsivity: dynamic food advertisements elicit stronger activation in regions critical for reward processing, including the amygdala and insula, compared with static images, indicating the heightened salience of multimedia marketing in engaging reward pathways (82). Collectively, these findings suggest that developing brains are particularly susceptible to food cues, with reward regions being easily engaged and inhibitory networks less effectively recruited, which may contribute to overeating and weight gain in vulnerable children.

However, the heterogeneity of age ranges studied—from early childhood to late childhood—makes it difficult to determine whether observed neural differences reflect developmental stage, weight status, or both. Longitudinal designs following children across developmental transitions are needed to clarify these trajectories.

### 7.2 Hormones and metabolism

The interaction between hormones and metabolism has a significant impact on children’s food choices, influencing their overall growth and health. Hormones play a key role in shaping children’s food choices by regulating appetite, satiety, and cravings. Insulin, ghrelin, leptin, and cortisol are hormones that regulate appetite and feelings of fullness, influencing food choices and energy consumption (2). Furthermore, dopamine reinforces desires for extremely appetizing foods by interacting with these hormones to stimulate the brain’s reward system.

Leptin, produced by fat cells, signals fullness, but leptin resistance in children with obesity can lead to overeating. It was discovered that cravings in children are associated with higher fasting levels, independent of body weight and adiposity (71). Although further research is needed to determine whether high levels cause or contribute to cravings, the data suggest a possible association between leptin resistance and cravings. An imbalance in the hunger hormone ghrelin may lead to a greater desire for foods high in energy, as it enhances reward-driven eating by increasing the motivation for highly palatable foods, even when individuals are already satiated (69).

It is well known that stress-induced elevated cortisol levels can lead to emotional eating and a predilection for comfort foods high in fat and sugar (83). It was found that stress can influence children’s eating behaviors, increasing unhealthy eating behavior from as early as 8–9 years old. However, it had no effect on eating behavior in younger children. Parental influence may explain these differences, as younger children tend to rely more heavily on family food choices. The findings also highlight concerns about stress-driven, unhealthy eating persisting into adulthood, increasing obesity risk (70). However, more high-quality research is needed to investigate the underlying psychological and physiological mechanisms and the long-term effects on children’s health.

Insulin, a key metabolic hormone, plays a crucial role in regulating glucose levels and energy storage (84). Through its interactions with other hormones related to appetite, it helps regulate hunger and satiety (85). Dysregulated appetite and a greater predilection for high-sugar, energy-dense foods may result from insulin resistance, which is frequently observed in obese children (72). However, more evidence is needed to support this finding.

Childhood is crucial for the development of obesity because of a combination of environmental and biological variables. Rising obesity rates are a result of sedentary lifestyles and diets high in processed, high-energy meals. Large quantities and external food cues may cause children with poor appetite regulation to overeat, leading to poor eating behaviors (47). Thus, this highlights the impact of obesity on appetite regulation and eating behaviors, illustrating how biological and environmental factors interact to induce poor dietary patterns.

### 7.3 Gut-brain axis and eating behavior

Eating behavior is regulated by balancing homeostatic (the body’s energy needs) and hedonic (pleasure-driven) mechanisms, as well as complex interactions within the gut-brain axis. Early-life microbiome perturbations that predispose to dysbiosis—especially antibiotics in infancy and cesarean delivery—have been associated with higher odds of overweight/obesity in childhood, reinforcing the developmental sensitivity of the gut–brain axis. Meta-analytic evidence links repeated antibiotic courses (86) in the first year(s) of life and cesarean birth (87) with later adiposity. However, it is unclear whether the direct effects of antibiotics and cesarean delivery on the gut microbiota mediate this association.

Mechanistically, disruptions in the gut microbiota (dysbiosis) can exacerbate appetite dysregulation by predetermining eating behavior toward hedonic rather than homeostatic mechanisms (21). The gut–brain axis regulates food intake through hormonal signaling, such as ghrelin, cholecystokinin, and leptin, which convey information about nutritional status to the brain. In contrast, fermentation of dietary fibers by beneficial gut bacteria produces short-chain fatty acids (SCFAs) that promote satiety and metabolic balance (88–91). Recent experimental work in animal models has further identified the role of the gut–brain axis in nutrient-specific preference. For example, Ray (92) demonstrated two distinct gut nutrient-detection systems: one selective for fat and another for sugar, fat, and amino acids. Notably, disruption of the fat-sensing pathway abolished mice’s preference for dietary fat, highlighting potential mechanistic targets for managing cravings. While these findings offer valuable insights into sugar and fat regulation, their translational relevance to humans, particularly children, remains unclear. The impact of dietary diversity and probiotic treatments on gut microbiota and neurocognitive outcomes in children remains poorly understood. Future long-term studies are necessary. These studies should combine microbiome profiles, gut hormones, SCFA dynamics, neural activation markers, and real-world eating habits. This approach will help clarify the mechanisms involved and determine the extent to which these effects are lasting and widespread.

Children’s brains engage in multiple areas when exposed to palatable food stimuli. Key regions include: the medial prefrontal cortex (mPFC), which is involved in inhibitory control and decision-making related to self-regulation of food intake; the orbitofrontal cortex (OFC), which evaluates food rewards and is associated with hedonic (pleasure-driven) eating; the striatum, which responds to appetizing foods and processes reward and motivation, reinforcing the urge to eat palatable items; the insula, which integrates the flavor and sensory aspects of food (taste, smell, texture) with internal hunger/fullness cues; the hippocampus, which contributes to food choices by forming memories of previous eating experiences and the pleasure associated with them; and the amygdala, which attaches emotional significance to food cues and can trigger emotional or impulsive eating. These regions form an interconnected reward network that is particularly reactive in children, helping explain why children may find certain foods especially irresistible.

## 8 Interplay between the contributing factors

**Figure 3:**
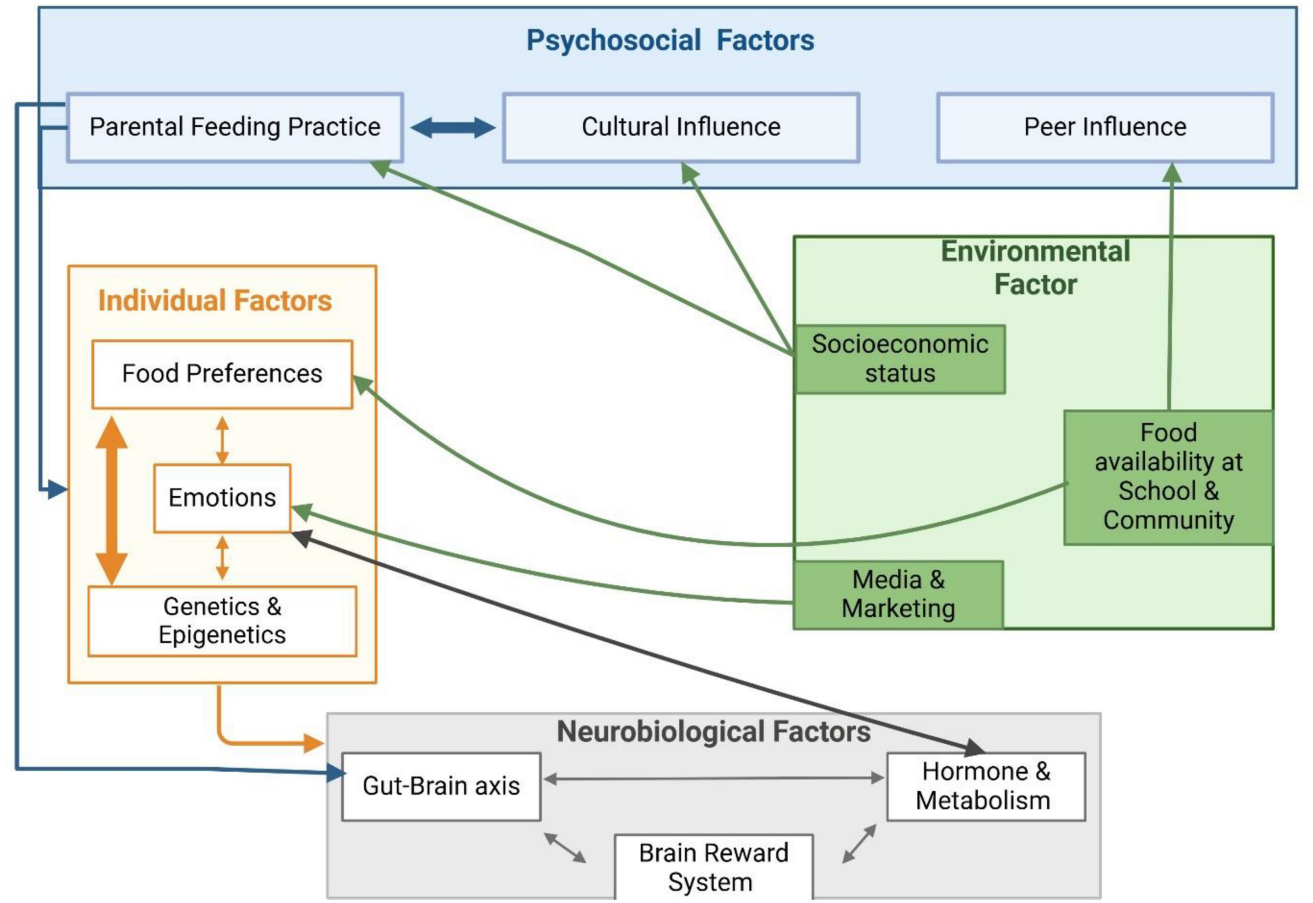
Hybrid model of children food choices’ drivers and their interactions. Created in BioRender. Hilary (110) (https://BioRender.com/uod3x46).

Children’s dietary choices are determined by a complex interplay of individual, neurobiological, environmental, and psychological factors (Figure 3). At the personal level, innate and learned food choices, emotions, and genetic and epigenetic factors greatly influence eating behaviors. The brain’s reward system is intimately related to children’s natural tastes for sweet and salty flavors, which likely evolved as a mechanism for recognizing foods high in energy (22) (108). These preferences are also shaped by the neurobiological activity of the dopamine system, which boosts the rewarding qualities of tasty foods, leading to a higher chance of overeating, particularly with high-calorie options (15, 109).

External environmental and psychosocial influences continuously shape this neurobiological predisposition. The availability of unhealthy meals in community and school settings, as well as targeted marketing and advertising strategies, play a crucial role in driving the preference for nutrient-poor, high-energy foods (20, 57). At the same time, peer and parental influences are crucial in changing or strengthening these preferences. While overly restrictive or permissive parenting styles can exacerbate unhealthy eating habits, positive parental actions, such as providing healthy food alternatives or setting an example of beneficial eating behaviors, can counteract these environmental influences (4, 44).

Emotions play a crucial role in connecting individuals and psychosocial factors. They affect how children react to both internal signals and outside triggers. When children feel negative emotions like stress or boredom, they often crave high-calorie, appealing foods (104). These eating habits are influenced by factors in their environment, such as food commercials (103, 104). On the other hand, healthy food choices can be promoted by positive emotional experiences associated with family meals or positive peer influences (48). Parents’ feeding approaches, such as emotional or restrictive feeding, can unintentionally encourage unhealthy habits, including emotional eating and overeating (47). Additionally, emotional eating has been associated with poor perception, alterations in the gut microbiota, and difficulties in recognizing emotions (35).

While genetic predispositions play a role in children’s appetitive traits, such as a preference for sweets and an aversion to bitter tastes, environmental factors, including parental feeding practices and the broader food environment, significantly influence dietary patterns (19). Epigenetic factors, which are influenced by maternal nutrition during pregnancy and early childhood exposures, provide a biological foundation that interacts with neurobiological, environmental, and psychosocial elements. For example, maternal adherence to a Mediterranean diet has been linked to healthier behavioral outcomes in children, while prolonged breastfeeding has been shown to enhance preferences for vegetables (28, 33), indicating that genetic influence is not deterministic. These early-life variables combine with psychosocial and environmental factors to shape long-term eating habits. However, age differences affect the relative relevance of influencing factors. For example, genetic factors may predominate during infancy due to poor cognitive override abilities (93). In contrast, peer influences become more powerful in later childhood as social cognition develops (48).

This intricate interplay shows how important it is to understand children’s food choices. Neurobiological mechanisms, environmental factors, and psychosocial dynamics shape and reinforce individual tendencies. Therefore, to develop effective interventions that promote healthier and more sustainable eating behaviors in children, there is a need for a comprehensive approach that considers these interconnected factors.

This diagram illustrates several areas that collaborate to influence eating behavior. On the right, individual factors include natural taste preferences, temperament, emotional states, and genetic or epigenetic traits. These characteristics influence how children perceive food. For instance, genetic taste sensitivities might make certain vegetables taste very bitter to some children but not others. They also affect how children respond to hunger and fullness signals.

The bottom layer discusses neurobiological influences, such as the brain’s reward system, which can lead to cravings for sugar or fat, the development of executive function, hormones like ghrelin and leptin, and the gut-brain axis, which encompasses signals from the microbiome that influence hunger and preferences. These biological factors shape the internal drives to eat and how rewarding a child finds different foods.

The top layer encompasses psychosocial factors, including family environment, which involves parental modeling and feeding practices, peer influence, and cultural norms. These elements provide the social context for forming eating habits. Environmental factors on the right side consist of external elements such as food availability and access at home, school, and in the community. Other influences include marketing and media exposure, socioeconomic conditions, and broader policies such as nutrition regulations and food laws. These factors affect the available food choices and those that are promoted. The model shows arrows that indicate bidirectional influences. For instance, individual preferences can affect how a child interacts with their environment. A picky eater might reject healthy food, even if it is available. On the other hand, the environment can shape preferences over time. Repeated exposure can lead to a stronger liking for certain foods. Psychosocial factors can also impact neurobiological responses. A supportive family environment can help lower stress and reduce stress-related eating behaviors.

Overall, the key message is that children’s food choices come from interactions among all these factors. No single area acts alone. Therefore, effective interventions should address multiple levels. These levels include nutrition education, family counselling, improved school food offerings, and a better understanding of appetite signals. Targeting these connected areas together can help guide children toward healthy eating habits.

## 9 Implications for nutrition recommendations, interventions, and policy development

Effective interventions require a multi-level approach that considers psychosocial, environmental, neurobiological, and individual determinants in interaction rather than isolation. They must balance feasibility, sustainability, and scalability while building on strategies already in place.

Family-based approaches remain central. Parents shape the home food environment, model eating behaviors, and influence children’s emotional relationships with food. Evidence shows that improving the availability of healthy foods at home (38), avoiding overly restrictive feeding practices (40, 46), and supporting responsive feeding styles can reduce overeating and improve acceptance of nutritious options (40, 45, 46). Maternal diet during pregnancy and lactation has long-term implications for children’s dietary patterns (18, 28). These strategies are highly feasible but require parental awareness, nutrition literacy, and support structures that are not always equally accessible across populations.

Schools provide a practical and scalable site for interventions. School-based programs were implemented to promote fruit and vegetable consumption, reduce intake of unhealthy snack (20, 55). National policies on school meals have improved children’s diet quality in several contexts. However, challenges like fast-food restaurants near schools and food marketing persist (54). Integrating media literacy and regulating unhealthy food advertisements, can enhance the impact of these strategies (34).

From a neurobiological perspective, interventions targeting reward processing, self-regulation, and metabolic balance can encourage healthier food choices. Programs that promote mindful eating and self-regulation training have shown benefits in children’s responses to hunger and satiety cues, in children’s control and impulsive eating (94). Additionally, physical activity, which enhances appetite regulation, hormonal balance and resilience against obesity-related brain changes, can complement dietary interventions (95, 96, 98, 99). While effective, these strategies require long-term reinforcement and integration into school and community programs for feasibility.

Policy and environmental interventions are powerful for long-term changes. Measures such as taxes on sugar-sweetened beverages and ultra-processed foods have led to reductions in consumption (66). National nutritional standards for school meals and financial support to improve affordability of fruits and vegetables are promising. The feasibility of such policies depends on political will, cross-sector collaboration, and industry engagement (100). Importantly, these measures make the healthy choice the easy, affordable, and convenient choice, which is crucial for long-term effectiveness.

## 10 Research gaps and future directions

Several limitations of the current evidence base should be acknowledged. Many studies are observational or cross-sectional, which restricts the ability to make causal inferences. Most studies examined these influences separately, rather than employing a combined approach that reflects their complex interactions. Much of the evidence comes from high-income countries, which limit their applicability to low- and middle-income settings, where cultural norms, socioeconomic conditions, and food environments differ significantly. Neurobiological studies often group wide age ranges together. This makes it challenging to distinguish between developmental effects and differences in weight status. Measurement issues also limit the findings. For example, eating behavior was primarily assessed through self-report questionnaires, which can be subject to bias. Many school-based interventions fail to control socioeconomic factors, despite their well-known impact on diet quality. Together, these limitations show the need for more thorough and diverse research designs.

Addressing these gaps requires several priorities for future research. First, there is a need for more longitudinal and experimental designs, including randomized controlled trials, to establish causal pathways between psychosocial, environmental, and biological influences on children’s food choices. Emphasis on investigations that examine the long-term effects of early nutritional experiences. This includes how maternal diet, the gut-brain axis, and food reward mechanisms influence children’s dietary paths. Second, multidisciplinary and integrative approaches should be adopted to capture the combined and interactive effects of these factors. Third, expanding research in low- and middle-income countries is essential to ensure that cultural and socioeconomic diversity is represented in the evidence base. Fourth, age-specific and developmentally sensitive designs to understand the roles of growth, puberty, and weight status in shaping eating behaviors should be considered. Fifth, improving objective measurement strategies, such as dietary biomarkers, neuroimaging, and digital tracking tools, will help address the limitations of self-reports. Finally, intervention research must systematically tackle socioeconomic disparities, ensuring that nutrition programs and school-based strategies are both fair and effective across diverse populations.

## 11 Conclusion

This review examines several factors that influence children’s dietary preferences. It considers individual, psychological, environmental, and neurological influences. Together, these elements shape what children choose to eat, often creating habits that last into adulthood. Family dynamics play a significant role in early eating habits, mainly through parental modelling and household environment. External influences, such as peer pressure, socioeconomic status, cultural norms, media exposure, and school and national policies, also impact dietary choices. Concurrently, biological factors, including brain development, reward circuits, metabolism, hormonal changes, the gut-brain axis, genetics, and epigenetics, significantly impact children’s food preferences and decisions. All these factors explain why some children prefer certain foods and why those habits can be hard to change.

By addressing these interconnected factors through coordinated interventions, researchers and policymakers can support environments that promote healthier, more sustainable dietary patterns in childhood. This approach will ultimately enhance long-term health outcomes and reduce the prevalence of diet-related diseases.
